# Medical Society of the County of Erie

**Published:** 1911-04

**Authors:** Franklin C. Gram

**Affiliations:** Secretary


### SOCIETY PROCEEDINGS

### Medical Society of the County of Erie

REPORTED BY FRANKLIN C. GRAM, M.D., Secretary,

THE regular meeting of the Medical Society of the County of Erie was held in the rooms of the Buffalo Society of Natural Sciences on Monday, February 20, 1911. President McClure was in the chair.

On motion, the reading of the minutes of the annual meeting was dispensed with as they had been printed in the Buffalo Medical Journal.

The secretary read the minutes of the council meetings held in the interim which, on motion, were approved and adopted.

The adoption of these minutes carried the resignations of Dr. T. H. Wilson, Buffalo; Dr. Harry Y. Grant, Falls View, Ont., and Dr. H. P. Frost, Boston, Mass.

Dr. Wall, chairman of the membership committee, presented the following list of applications for membership, and the secretary was directed to cast the ballot of the society for their election, all the physicians being residents of Buffalo, N. Y.

Guy B. Crandall, 1407 Fillmore avenue; Henry J. Doll, 1124 Genesee street; George P. McCarthy, 749 Fillmore avenue; Edward F. Healy, 444 Elk street; Aron M. Swados, 392 Amherst street; George W. Puerner, 385 Walden avenue; Edward D. Clark, 12 Parker avenue; Edward Durney, 208 Baynes street.

Dr. Hopkins, chairman of the committee on public health, presented a resolution and moved its adoption as follows:

The Medical Society of the County of Erie believes that a new school for truants is a necessity. We believe that in the selection of a site the interests of the boys should receive first consideration and in view of this we believe that in location and equipment the school should represent the best thought of the country. The school should provide for at least 200. It should be at least twelve miles from the city line. There should be a stream of water flowing through the place. It should be a place of such scenic beauty as to have a transforming power on the lives of the young people who are to live there. It should be at least a mile from railroad or trolley connections. We earnestly petition those in authority to give due consideration to these principles.

The resolution was adopted.

Dr. Blaauw called up the question of division of fees, which had been before the society at a previous meeting in which it was stated that the chief fault was the overcrowding of the profession, and suggested that pamphlets be published on the subject to be distributed in the high schools. No action was taken.

A communication was received from the Chemung County Medical Society in which it was stated that that society had adopted a resolution asking the State Society to discontinue the publication of the State Medical Directory.

A communication accompanied by resolutions was received from the Medical Society of the County of New York taking an exact opposite view and requesting the continuance of the publication in question.

On motion of Dr. Grosvenor, the delegates of the Medical Society of the County of Erie to the State Society were instructed to oppose the discontinuance of the State Directory.

Dr. Hartwig asked why publish the names from other states and not confine the directory to New York State.

Dr. Wall explained that the income from the sale and advertising is sufficient to more than offset the additional expense and because those states desire and depend upon their representation in the directory.

This concluded the regular order of business, after which the scientific program was presented as follows: "A plea for the early operative treatment of rectal diseases," by Edward Clark; "Some indications for the use of blood serum and for the use of blood transfusion," by F. C. Busch; "Double inguinal strangulated hernia—cryptorchid—operation," by L. G. Hanley; "Report of cases of heart-block and Stokes-Adams syndrome," by John Gray; "Differential diagnosis of spinal diseases," by F. S. Crego. Discussion followed the reading of above papers, after which a collation was served.

